# Recent Advances in Microfluidic Chip Technology for Laboratory Medicine: Innovations and Artificial Intelligence Integration

**DOI:** 10.3390/bios16020104

**Published:** 2026-02-05

**Authors:** Hong Cai, Dongxia Wang, Yiqun Zhao, Chunhui Yang

**Affiliations:** Department of Clinical Laboratory, The Second Hospital of Dalian Medical University, Dalian 116023, China; hongcai_0706@dmu.edu.cn (H.C.); nxbcehow-33@dmu.edu.cn (D.W.)

**Keywords:** microfluidic chip technology, laboratory medicine, artificial intelligence, point-of-care testing

## Abstract

Microfluidic chip technologies, also known as lab-on-a-chip systems, have profoundly transformed laboratory medicine by enabling the miniaturization, automation, and rapid processing of complex diagnostic assays using minimal sample volumes. Recent advances in chip design, fabrication methods—including 3D printing, modular and flexible substrates—and biosensor integration have significantly enhanced the performance, sensitivity, and clinical applicability of these devices. Integration of advanced biosensors allows for real-time detection of circulating tumor cells, nucleic acids, and exosomes, supporting innovative applications in cancer diagnostics, infectious disease detection, point-of-care testing (POCT), personalized medicine, and therapeutic monitoring. Notably, the convergence of microfluidics with artificial intelligence (AI) and machine learning has amplified device automation, reliability, and analytical power, resulting in “smart” diagnostic platforms capable of self-optimization, automated analysis, and clinical decision support. Emerging applications in fields such as neuroscience diagnostics and microbiome profiling further highlight the broad potential of microfluidic technology. Here, we present findings from a comprehensive review of recent innovations in microfluidic chip design and fabrication, advances in biosensor and AI integration, and their clinical applications in laboratory medicine. We also discuss current challenges in manufacturing, clinical validation, and system integration, as well as future directions for translating next-generation microfluidic technologies into routine clinical and public health practice.

## 1. Introduction

Microfluidic chip technologies—often known as lab-on-a-chip systems—have revolutionized laboratory medicine by miniaturizing and automating complex assays on tiny platforms [1,2]. By precisely manipulating nanoliter to microliter volumes of fluids in networks of microscale channels, valves, and chambers, microfluidics enables rapid, high-throughput analysis of biological samples with minimal reagents and sample volumes. This capability has already transformed biosensing and diagnostics, allowing detection of biomarkers with enhanced sensitivity, speed, and cost-efficiency compared to conventional benchtop assays [3]. As a result, microfluidic devices are emerging in diverse clinical applications—from POC infectious disease tests to sophisticated liquid biopsy platforms for cancer [4].

Laboratory medicine is facing increasing demands for faster diagnostics and personalized monitoring, amid workforce limitations and rising test volumes [5]. Microfluidic innovation offers a timely solution: highly automated, portable, and accessible diagnostic platforms that can deliver lab-quality results at the bedside or in resource-limited settings. These chips integrate sample preparation, biochemical reactions, and signal detection into a single miniaturized device, dramatically accelerating turnaround times for critical tests (often minutes instead of days) and reducing human error through automation [6,7]. In recent years, the field has seen a surge of new designs and fabrication techniques that improve device performance, reliability, and manufacturability [8]. Researchers have introduced advanced materials and fabrication methods—including 3D printing, paper-based substrates, and modular components—that simplify production and enable more complex on-chip functionalities [9,10,11]. Concurrently, there is growing integration of biosensors (optical, electrochemical, etc.) directly into microfluidic chips to provide sensitive real-time readouts of analytes, from metabolites and proteins to nucleic acids [12,13].

A transformative trend in recent years is the convergence of microfluidics with AI and machine learning [14]. AI algorithms are being deployed to enhance microfluidic platforms at multiple levels: optimizing chip designs, controlling fluidic operations, and analyzing the complex data or images produced by on-chip assays [15,16]. This synergy enables “smart” lab-on-chip systems that can self-correct errors, interpret results automatically, and even support clinical decision-making by comparing outputs to vast training datasets [17,18]. The integration of microfluidics, automation, and AI is positioning these technologies as adaptable solutions for highly efficient and personalized medicine, capable of tailoring diagnostics and treatments to individual patient data [19].

**Figure 1:**
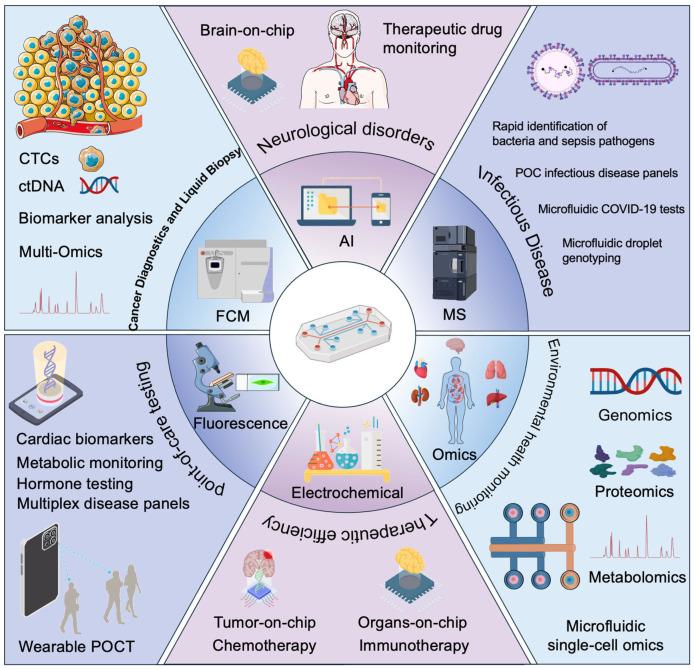
Overview of recent advances and clinical applications of microfluidic chip technologies in laboratory medicine. Microfluidic platforms are enabling breakthroughs across cancer diagnostics (e.g., CTC and ctDNA analysis, multi-omics liquid biopsy), point-of-care testing (including wearable POCT, cardiac and metabolic biomarker assays), therapeutic efficiency (such as tumor-on-chip drug screening and organs-on-chip immunotherapy), infectious disease detection (rapid pathogen identification, COVID-19 testing), neurological and therapeutic monitoring (brain-on-chip, real-time drug monitoring), and environmental health monitoring (genomics, proteomics, metabolomics, single-cell omics). Integrated detection modalities—including fluorescence, electrochemical sensors, flow cytometry (FCM), and mass spectrometry (MS)—alongside artificial intelligence (AI), further enhance microfluidic chip performance, automation, and data interpretation for personalized and precision medicine.

This review provides a comprehensive overview of recent developments in microfluidic chip technologies for laboratory medicine. We begin by describing innovations in microfluidic chip design, fabrication, and biosensor integration that have enhanced device capabilities. We then examine the primary clinical applications of these chips—including cancer diagnostics, infectious disease testing, POC devices, personalized medicine approaches, and therapeutic monitoring–highlighting representative studies and real-world implementations (Figure 1). Next, we discuss the critical role of AI integration in advancing microfluidic platforms, from automated image analysis to predictive analytics and decision support. We also survey emerging application areas such as neuroscience diagnostics, microbiome profiling, and environmental health monitoring, where microfluidics is opening new frontiers. Finally, we address the challenges and future directions for translating these technologies into mainstream clinical practice, including issues of regulatory approval, standardization, and the need for interdisciplinary innovation. By synthesizing high-impact research and clinical trials from the past five years, this review illuminates how microfluidic chip technologies–increasingly augmented by AI—are transforming laboratory medicine and what promises and hurdles lie ahead.

## 2. Recent Innovations in Microfluidic Chip Design and Fabrication

**Figure 2:**
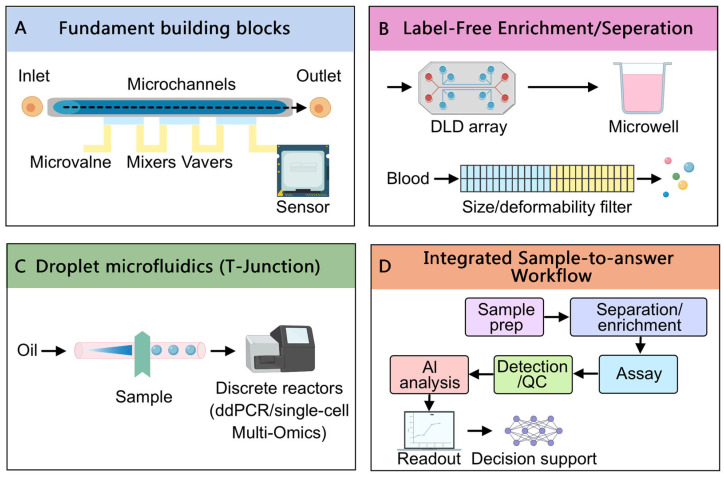
Core microfluidic unit operations and representative architectures relevant to laboratory medicine. (**A**) Fundamental building blocks within microfluidic chips (inlet/outlet, microchannels, mixers, valves, and an on-chip detection window). (**B**) Representative label-free enrichment/separation layouts (deterministic lateral displacement (DLD) pillar arrays, inertial spiral channels, and size/deformability-based filtration) used to improve target purity before downstream assays. (**C**) Droplet microfluidics at a T-junction for compartmentalizing reactions (e.g., ddPCR and single-cell assays) into discrete droplets. (**D**) An integrated sample-to-answer workflow linking sample preparation, enrichment, on-chip assay, detection/quality control, and AI-assisted analysis/decision support.

Microfluidic device engineering has advanced rapidly in the last five years, yielding new chip designs and fabrication methods that improve performance, manufacturability, and integration of biosensors (Figure 2). Traditional microfluidic chips were often fabricated in polydimethylsiloxane (PDMS) using soft lithography, which provides excellent precision and biocompatibility but can be labor-intensive and difficult to scale [20]. Recent innovations have focused on materials and fabrication techniques that overcome these limitations.

### 2.1. Three-Dimensional Printing and Additive Manufacturing

Additive manufacturing has emerged as a game-changer for prototyping and producing microfluidic structures. Stereolithography (SLA) and other 3D printing methods can directly create intricate microchannel networks and custom geometries in a single step [21]. Unlike photolithography (which requires cleanroom facilities and masks), 3D printing offers design flexibility, rapid iteration, and reduced fabrication time [22]. Researchers have leveraged high-resolution printers with transparent, biocompatible resins to fabricate complex chips, such as multi-layer microfluidic circuits and organs-on-chips, that would be challenging to produce via conventional molding [23]. These 3D-printed microfluidic platforms can incorporate features like interconnected channel networks, valves, and reservoirs without extensive assembly. For example, Deliorman et al. (2023) [7] highlight that 3D printing enables rapid creation of microchannels and even 3D tissue scaffolds within chips, accelerating drug development and disease modeling experiments. While print resolution (feature size) is still coarser than photolithography for sub-10 µm structures, ongoing improvements in printable materials and printer precision are closing this gap [24]. For instance, standard photolithography-based soft lithography can routinely produce channel features just a few micrometers in size, whereas most commercial 3D printers are currently limited to minimum feature sizes on the order of tens of micrometers (~10–50 µm). This difference means that capturing single cells (~10–20 µm diameter) or subcellular vesicles often requires photolithographic fabrication for precise structures (ensuring only one cell is captured per trap or droplet), while 3D-printed devices might struggle with such fine isolation unless cutting-edge high-resolution methods are employed. In clinical terms, insufficient resolution could lead to lower purity in single-cell capture—for example, a 3D printed cell trap might inadvertently hold multiple cells or allow a target cell to escape, impacting assay sensitivity. Notably, 3D printing is especially attractive for rapid prototyping of new chip designs, where iterating on a design no longer requires weeks of mold fabrication but can be done in hours [25]. Recent advances (e.g., high-resolution LCD-based printers) have demonstrated features approaching ~10 µm, but photolithography remains the gold standard for sub-10 µm features critical to single-cell work.

### 2.2. Modular and Lego-like Microfluidics

A significant new concept is modular microfluidic design, which uses interchangeable building blocks to assemble fluidic circuits. Inspired by the famous brand LEGO bricks, injection-molded microfluidic modules with standardized connectors have been developed that snap together to form complex lab-on-chip systems [26]. This modularity allows users to reconfigure chips for different functions (mixing, cell sorting, filtration) simply by rearranging components, greatly enhancing versatility and easing prototyping. Researchers at MIT demonstrated a Lego-like microfluidic platform of plug-and-play blocks that performed operations like blood cell separation and chemical gradient generation when joined in various sequences [27]. Such modular systems address standardization issues by ensuring each piece fits reliably, and they facilitate scaling up production via mass-molded plastic parts. The past few years have seen increased availability of commercial modular microfluidic kits, which lowers the barrier for laboratories to build custom devices without specialized fabrication facilities [28].

### 2.3. Paper-Based and Flexible Microfluidics

To enable ultra-low-cost, POCT, engineers have advanced paper microfluidic devices (also called μPADs) that channel fluids through porous paper via capillary action [29,30]. Recent work has improved the patterning of hydrophobic barriers on paper (using wax printing, inkjet etching, etc.) to create more complex circuits for multistep assays [31,32]. Paper-based chips are attractive because they are inexpensive, disposable, and require no external pumps (fluid wicks by capillarity). Paper microfluidics were widely applied to infectious disease diagnostics in low-resource settings—for example, origami-style folded paper devices that perform enzyme linked immunosorbent assay (ELISA) or nucleic acid amplification and show colorimetric results [33,34,35]. Flexible microfluidic substrates (like polyester films or elastomers) have also enabled wearable microfluidics [36], such as skin patches that collect sweat for analysis of electrolytes and metabolites on-site [37]. These trends broaden the reach of microfluidics beyond rigid chips toward conformable, field-deployable formats.

### 2.4. Advanced Material Microfabrication

Traditional materials like silicon and glass are being complemented by new polymers and composites. Rigid thermoplastics (e.g., Polymethyl Methacrylate (PMMA), copolymers of cycloolefin (COC)) can be mass-produced via injection molding or hot embossing, yielding low-cost disposable chips for clinical use [38]. This is especially useful for manufacturing cartridges for blood analyzers and molecular testing—once a mold is made, thousands of identical plastic chips can be stamped out with excellent reproducibility. Another innovation is integrating hydrogels and porous media inside chips to better mimic tissue environments (for cell culture) or to filter/separate analytes [39]. For instance, microfluidic organ-on-chip devices now often include extracellular matrix gels patterned in microchannels to support 3D cell growth (vital for personalized drug testing, discussed later) [40]. Additionally, hybrid fabrication approaches have emerged: one can 3D-print a rough fluidic device and then micro-mill or emboss fine features where high precision is needed, combining speed with accuracy.

Alongside fabrication advances, microfluidic design principles have matured. Computational fluid dynamics and even machine learning optimization are now used to refine channel layouts, ensuring uniform flow distribution and efficient mixing/reaction on-chip [41]. Machine learning models can predict how subtle geometry changes impact flow or cell capture efficiency, allowing automated design optimization before a chip is ever built [42]. This has given rise to more complex architectures such as droplet microfluidics [43] and digital microfluidics [44]. Droplet microfluidics—generating picoliter droplets as isolated reaction vessels—was already a hot area, but recent designs allow individual control of each droplet (“digital” control) via electrode arrays (electrowetting) [45]. This digitization of microfluidics turns biochemical processes into sequences of electrically controlled droplet operations (splitting, merging, mixing), enhancing programmability [46]. Up to now, digital microfluidic platforms have been used for multi-step clinical assays where the device software directs droplets through all assay steps automatically, much like a “lab-on-a-chip” executing a protocol [47].

Crucially, biosensor integration into microfluidic chips has improved the functionality and readout of these systems. Modern microfluidics are often coupled with on-chip sensors that transduce chemical or biological events into measurable signals.

#### 2.4.1. Optical Sensors

Many chips incorporate optical detection zones—e.g., tiny chambers where fluorescence or color change is measured. LED and photodiode components can be embedded or aligned with the chip to detect signals from immunoassays or DNA amplifications [48]. Advanced examples include guided-mode resonance sensors or plasmonic nanostructures built into microchannels for real-time monitoring of binding events, achieving very low detection limits (such as detection of Alzheimer’s biomarker proteins via a nanoplasmonic sensor in a microfluidic flow cell) [49].

#### 2.4.2. Electrochemical Sensors

Thin-film electrodes (gold, carbon, etc.) can be printed or deposited inside microchannels to directly measure analytes via electrochemical reactions. Recent devices use arrays of microelectrodes to detect metabolites, ions, or DNA on chip with high sensitivity [50,51]. For instance, integrated electrochemical biosensors on microfluidic chips have been developed for biofilm monitoring and antibiotic drug detection [52]. The tight integration shortens analysis time by eliminating off-chip sample transfer; an example is a microfluidic ELISA with on-chip electrodes that quantifies the enzymatic product immediately in the flow, speeding up immunodiagnostics to just a few minutes [53].

#### 2.4.3. Physical and Other Sensors

MEMS pressure sensors [54], temperature sensors [55], or even acoustic transducers [56] are being embedded to closely control and read conditions within chips. The result is self-contained analytical microsystems that not only carry out reactions but also measure and regulate them in real time.

In summary, microfluidic technology has matured from simple PDMS channel devices to sophisticated, integrated analytical machines. Advances in materials (3D-printable polymers, paper, thermoplastics), fabrication (modular design, large-scale molding, hybrid printing), and on-chip sensors have greatly expanded what microfluidic chips can do. Standardization efforts, like modular blocks and consistent fabrication protocols, are addressing reproducibility and scalability, aiming to transition microfluidics from the research bench to widespread clinical and commercial use. These innovations lay the groundwork for the diverse clinical applications discussed in the next section, and they provide a solid platform for incorporating AI to further enhance chip capabilities.

## 3. Clinical Applications of Microfluidic Platforms

Microfluidic chip technologies are now being applied across a broad range of clinical and diagnostic domains. Their ability to rapidly process small samples and provide accurate, real-time results has driven innovation in numerous areas of medicine compared to traditional detection methods (Table 1). Here we highlight several primary applications in laboratory medicine: cancer diagnostics (especially liquid biopsy), infectious disease testing, POCT, personalized medicine, and therapeutic drug monitoring [57,58,59]. In each area, microfluidics offers unique advantages for detecting biomarkers and guiding clinical decisions (Table 2).

### 3.1. Cancer Diagnostics and Liquid Biopsy

Microfluidic devices have become central to liquid biopsy approaches, which involve detecting tumor-derived biomarkers (such as CTCs, DNA, or exosomes) in body fluids like blood, enabling minimally invasive cancer diagnostics [60]. Microfluidic biosensors have proven exceptionally adept at isolating and analyzing these rare biomarkers, offering high sensitivity and specificity in capturing targets that would be missed by bulk methods.

#### 3.1.1. CTCs

Microfluidic devices are at the forefront of CTC isolation from blood. Capturing CTCs is challenging—they are extremely rare (often a few cells per billion blood cells)—but microfluidic chips can be engineered with precise geometries and surface properties to selectively trap CTCs while letting normal blood cells flow through [61]. For example, spiral microfluidic devices with size-based separation have been refined: a 2023 study by Seyfoori et al. developed a bilayer microfluidic chip using a smart antibody-coated magnetic hydrogel; as blood flowed through, CTCs bound to the magnetic gel and were efficiently separated under a magnetic field [62]. Another group designed a trapezoidal-spiral microchannel that exploits Dean flow and inertia to focus CTCs, achieving ~90% purity and recovery of cancer cells at clinically relevant flow rates [63]. Such chips yield enriched CTC populations that can be stained, counted, and genetically analyzed on-chip or off-chip.

In recent years, several microfluidic-based CTC detection platforms have been approved by the U.S. Food and Drug Administration (FDA) for clinical use. Among these, the CellSearch^®^ system is the most representative, initially receiving FDA approval in 2004 for detecting CTCs in metastatic breast cancer patients and subsequently gaining approval for prostate and colorectal cancers [64]. Although the CellSearch system employs immunomagnetic bead-based approaches for CTC enrichment, newer microfluidic chip technologies have further enhanced sorting accuracy and purity. For example, the ClearCell^®^ FX system, based on inertial microfluidics, has recently entered clinical evaluation. This device utilizes specially designed spiral microchannels that exploit cell-size differences and Dean flow effects to rapidly and efficiently enrich CTCs. Studies have demonstrated capture efficiencies exceeding 80%, with excellent preservation of cell viability, enabling downstream genomic and proteomic single-cell analyses [65].

Different CTC isolation strategies offer trade-offs between purity and recovery. Immunoaffinity-based capture (using antibodies against epithelial markers such as EpCAM, as in the CellSearch^®^ system) tends to yield very high purity since only antigen-positive cells are retained, but it may miss CTCs that have downregulated the target antigen (lowering recovery). Label-free approaches, such as size-based filtration or inertial focusing, capture a broader spectrum of CTCs (including mesenchymal or EpCAM-negative cells) and often achieve high recovery (>80–90% of spiked tumor cells), but they typically co-isolate some larger leukocytes, resulting in lower purity [66]. For example, inertial spiral microdevices can enrich CTCs at high throughput but may require secondary steps to remove white blood cells in the ~15–20 µm size range that overlap with CTC size, to improve purity [67]. Recent systems combine approaches: the CTC-iChip developed by Harvard University integrates inertial microfluidics with immunoaffinity-based negative selection, using deterministic lateral displacement (DLD) to remove small cells and then magnetically depleting labeled leukocytes. This label-free, high-throughput process yields highly efficient isolation of CTCs directly from whole blood [68]. Concurrently, the FDA granted approval in 2022 for ANGLE’s Parsortix^®^ microfluidic system for clinical analysis of CTCs. This device employs a unique microstructure design enabling precise, label-free capture of intact CTCs (by physically trapping larger, less deformable cells) suitable for subsequent molecular analyses, thus providing critical information for early cancer diagnosis, prognosis, and therapeutic monitoring [69]. In summary, microfluidic CTC chips can be tuned for maximum recovery (capturing as many CTCs as possible) or high purity (minimizing WBC carryover), and modern designs strive to optimize both metrics by leveraging physical properties (size, deformability) alongside specific binding as needed.

Another noteworthy innovation is the CTC-iChip developed by Harvard University, which combines inertial microfluidics with immunoaffinity-based approaches for the label-free, highly efficient isolation of CTCs directly from whole blood. Recent preclinical studies have validated the high sensitivity of the CTC-iChip in detecting CTCs from blood samples of patients with non-small cell lung cancer, breast cancer, and melanoma, positioning it as a promising next-generation clinical liquid biopsy platform [70]. Concurrently, the FDA granted approval in 2022 for ANGLE’s Parsortix^®^ microfluidic system for clinical analysis of CTCs in ovarian cancer patients [71]. This device employs a unique microstructure design enabling precise, label-free capture of intact CTCs suitable for subsequent molecular analyses, thus providing critical information for early cancer diagnosis, prognosis evaluation, and therapeutic monitoring.

Microfluidic chip technology not only improves the capture efficiency and detection sensitivity of CTCs but also facilitates the advancement of single-cell analyses and precision medicine. In recent studies, microfluidic platforms combined with single-cell sequencing technologies have enabled, for the first time, real-time genomic and transcriptomic profiling of CTCs. For instance, a landmark 2024 study published in Nature Communications described an integrated microfluidic chip capable of fully automated cell capture and lysis, achieving comprehensive single-cell transcriptomic sequencing of CTCs directly from blood samples. This integrated approach provides immediate gene expression profiles, guiding targeted therapeutic strategies [72,73].

**Table 2:** Key microfluidic chip applications in modern laboratory medicine.

Clinical Application	Detected Biomarkers/Substances	Detection Method	Special Microfluidic Techniques	References
Cancer Diagnostics (Liquid Biopsy)	CTCs	Immunoaffinity, inertial microfluidics	Spiral microchannels, Dean flow separation, magnetic hydrogels	[1,2,3,4,64]
	ctDNA	ddPCR, CRISPR-based detection, SERS	Digital droplet microfluidics, finger-powered chip, CRISPR-Cas integration	[74,75,76,77]
	Exosomes	Immunoaffinity, Raman spectroscopy	Staggered micropillar arrays, nanoporous filtration, acoustic or electric enrichment	[77,78,79]
Infectious Disease Testing	SARS-CoV-2 RNA, respiratory viruses	RT-PCR, RT-LAMP, CRISPR-Cas	Centrifugal lab-on-disc, multiplex channels, lateral-flow integration	[80,81,82]
	Bacteria (Antibiotic susceptibility)	Optical, electrochemical detection	Single-cell droplet microfluidics, rapid AST, digital droplet assays	[83,84]
POCT	Cardiac biomarkers (Troponin I, NT-proBNP, CK-MB, D-dimer)	ELISA, electrochemiluminescence, fluorescence	Integrated valves/micropumps, digital immunoassay, spiral microfluidics	[85,86,87]
	Metabolic indicators (Glucose, HbA1c, Creatinine)	Electrochemical biosensors	Gold nanoparticles, carbon nanotubes, integrated enzymatic assays	[88]
	Pregnancy/hormones (Progesterone, LH, FSH)	Optical/colorimetric immunoassays	Multi-chamber microfluidics, smartphone imaging, integrated ELISA	[89]
	Multiplex infectious disease panels (Malaria, Dengue, Typhoid)	ELISA, PCR-based amplification	Multiplexed channels, parallel microreactions, integrated amplification assays	[18,82]
Personalized Medicine	Tumor drug response (Chemo-sensitivity tests)	Cell viability assays, fluorescence microscopy	Organ-on-chip, patient-derived tumor organoids, digital microfluidics	[90,91]
	Therapeutic drug monitoring (Tacrolimus, antibiotics)	Colorimetric immunoassay, electrochemical	Paper-based vertical-flow immunoassay, wearable patches	[92,93]
Integration with AI	Disease classification, anomaly detection	Deep learning image analysis, signal processing	Smartphone-based AI analysis, deep learning algorithms	[17,18,94,95]

#### 3.1.2. Microfluidic Detection of ctDNA and Exosomes in Liquid Biopsy

Microfluidic lab-on-a-chip platforms have achieved remarkable progress in recent years in analyzing acellular tumor markers like circulating tumor DNA (ctDNA) and exosomes. By integrating sample preparation with highly sensitive molecular assays on-chip, these systems can isolate and detect ctDNA from plasma with minimal hands-on steps. For instance, droplet digital PCR (ddPCR) microfluidics are now widely used in clinical laboratories to quantify ctDNA with high sensitivity and absolute accuracy [74]. Beyond conventional amplification, researchers have developed fully integrated chips that combine on-chip plasma filtration, target enrichment, and nucleic acid detection. One recent example is a finger-powered microfluidic chip that rapidly processes whole blood and employs a SERS-based assay (surface-enhanced Raman spectroscopy) to detect EGFR gene mutations in ctDNA without PCR amplification [75]. This portable device achieved an ultrasensitive limit of detection around 100 fM for mutant ctDNA in under 35 min, illustrating how microfluidics can enable amplification-free mutation screening at the point of care. Likewise, CRISPR-powered microfluidic biosensors have emerged for ctDNA analysis—for example, coupling recombinase polymerase amplification with Cas12a detection on-chip has allowed multiplexed identification of tumor DNA targets with attomolar sensitivity in under an hour [76]. Such integrated microfluidic systems minimize sample loss and dilution, enhancing detection of low-abundance tumor DNA that might be missed by bulk methods.

Parallel advances in microfluidics have greatly improved the isolation and analysis of tumor-derived exosomes, which are 30–150 nm extracellular vesicles carrying tumor proteins, DNA, and RNAs. Microfluidic chips can enrich these vesicles from serum using nanoporous filters, microvortices, or immunoaffinity capture, all within a small-footprint device. One innovative platform uses a staggered micropillar array coated with anti-CD63 antibodies to selectively trap exosomes on-chip [78]. In this device, the captured vesicles are tagged with EpCAM-functionalized Raman nanoparticles, enabling on-chip fluorescence or Raman readout of tumor-derived exosomes within an hour. Notably, the limit of detection reached ~160 exosomes per mL (using just a 20 μL sample) in clinical serum, demonstrating ultra-sensitivity in isolating scant vesicles. Other microfluidic techniques employ acoustic waves or electric fields to enrich exosomes based on size and dielectric properties, often in a label-free manner, to preserve vesicle integrity [96]. By processing small volumes under controlled micro-scale flows, these chips avoid the dilution and losses of traditional ultracentrifugation, concentrating nanometer-sized vesicles for analysis. The ability to retrieve intact exosomes carrying tumor biomarkers (e.g., oncogenic RNAs or surface proteins) is invaluable—studies have shown that exosomal cargo reflects the tumor’s molecular profile and can indicate disease state or treatment response [79]. Microfluidic exosome assays, therefore, offer a highly sensitive and noninvasive approach for early cancer diagnosis, potentially detecting tumor-derived vesicles even in early-stage disease when bulk methods yield little signal.

These next-generation microfluidic chips are designed with an emphasis on integration, multifunctionality, and throughput—all crucial for clinical liquid biopsy applications in laboratory medicine. Modern devices often incorporate multiple functional modules (cell removal, target capture, washing, and detection) on a single chip, creating an automated workflow. This integration not only shortens processing time but also improves analytical sensitivity by keeping analytes in a confined microenvironment. Many platforms are self-contained and automated; for example, pump-free designs using capillary action or vacuum-driven membranes allow operation without bulky equipment [75], and on-chip valves/mixers automate multi-step reactions. High-throughput capabilities are achieved by microfluidic parallelization and multiplexing. Researchers have demonstrated chips with arrays of microchambers or droplets to perform thousands of reactions in parallel, enabling digital PCR quantification of rare mutant DNA alleles with variant fractions below 0.1% [74]. In another approach, a high-throughput microfluidic fluidized bed was developed to rapidly process larger volumes of plasma for ctDNA extraction, using a dynamic mixture of magnetic beads to capture target sequences at flow rates up to 15 μL/min [77]. This greatly accelerates sample processing compared to traditional micro-scale flows and preconcentrates DNA for downstream analysis. Moreover, microfluidic platforms now allow multiplexed detection of numerous biomarkers in one run—a recent CRISPR-based chip simultaneously screened 30 different nucleic acid targets, highlighting the potential for comprehensive mutational profiling in a single test [76]. Overall, the ultrasensitive detection and automation afforded by microfluidic ctDNA and exosome assays are advancing the utility of liquid biopsies in clinical diagnostics. They enable earlier cancer detection and real-time monitoring of tumor dynamics through minimally invasive blood tests, which is transforming laboratory medicine by providing clinicians with actionable molecular insights without the need for invasive tissue biopsies.

### 3.2. Infectious Disease Diagnostics

The COVID-19 pandemic (2020–2022) underscored the critical need for rapid, accurate infectious disease diagnostics—a need that microfluidic technologies are well-positioned to meet. In the past five years, microfluidic chips have been extensively developed for detecting pathogens (viruses, bacteria) and their biomarkers at the point of care. These devices minimize sample handling and integrate complex processes (like nucleic acid amplification or serology) into self-contained cartridges, enabling fast diagnosis of infections in clinics or even at home.

#### 3.2.1. Microfluidic COVID-19 Tests

A flurry of microfluidic diagnostic kits for SARS-CoV-2 emerged during the pandemic. Many leveraged centrifugal microfluidics (lab-on-a-disc systems): fluidic cartridges that, when spun, use centrifugal force to move samples through reagent chambers for RNA extraction, amplification, and detection [97]. A systematic review in 2022 found that polymer microfluidic devices (largely PDMS or PMMA) were widely used in COVID tests, with centrifugal flow control being the most common technique, followed by capillary-driven flow in paper-based chips [2]. For example, a 2021 study reported an integrated centrifugal microfluidic platform performing reverse transcription loop-mediated isothermal amplification (RT-LAMP) of viral RNA from a nasal swab, yielding a colorimetric readout in under 30 min [80]. Another approach combined microfluidics with lateral flow strips: one team developed a microfluidic-integrated lateral flow assay where the chip automated mixing of sample and reagents for recombinase polymerase amplification (RPA) of SARS-CoV-2 RNA, then flowed the product onto a lateral flow strip for visual detection [81]. These systems achieved sensitivities comparable to RT-PCR but in a fraction of the time and without bulky instruments. Microfluidics also facilitated multiplexed COVID testing—e.g., chips that could test one sample for SARS-CoV-2 and influenza simultaneously by splitting the sample into parallel microchambers with different primers.

Beyond amplification tests, microfluidic immunoassays were created for viral antigen and antibody detection. Researchers in 2022 built chips that could detect SARS-CoV-2 antigens in nasopharyngeal samples using on-chip ELISA with electrochemical or optical readouts, providing an alternative to conventional lateral flow rapid tests [98]. Thanks to reduced diffusion distances and automated reagent handling, microfluidic immunoassays often showed improved analytical sensitivity over traditional rapid tests. Collectively, the pandemic catalyzed a leap in microfluidic POC diagnostics, and many of these technologies are now being extended to other infectious diseases.

#### 3.2.2. Rapid Identification of Bacteria and Sepsis Pathogens

Microfluidics has also advanced the diagnosis of bacterial infections, where time is critical for guiding therapy. Traditionally, identifying bacteria and their antibiotic susceptibility can take 1–3 days using cultures. Microfluidic systems have been designed to accelerate pathogen detection and antibiotic susceptibility testing (AST) dramatically [83]. For example, single-cell microfluidic AST chips partition blood or urine samples into arrays of tiny chambers, each containing one or a few bacteria with a specific antibiotic concentration. Bacterial growth (or death) in each chamber is monitored (optically or electrochemically) within hours, allowing determination of the minimum inhibitory concentration (MIC) of antibiotics much faster than macroscopic culture [99]. Zhang et al. (2020) reviewed such microfluidics-based AST devices, noting that by confining bacteria in microdroplets or narrow channels, one can observe phenotypic responses to drugs at the single-cell level—revealing antibiotic resistance within hours [83]. One innovative AST platform uses a digital microfluidic device to combine a bacterial sample with a panel of antibiotics in nanoliter droplets; machine vision then detects bacterial growth in each droplet via fluorescence, completing a full antibiotic susceptibility profile in ~4–5 h [84]. These rapid AST tools are crucial for sepsis management, where every hour of delay in appropriate antibiotics increases mortality—a microfluidic AST that guides therapy within the same clinical shift can be life-saving.

Additionally, microfluidic chips are enabling sample-to-answer detection of bacteria from complex samples (blood, stool) without culture. For instance, a fully automated microfluidic PCR cartridge (the size of a credit card) was developed to extract DNA from blood, amplify bacterial 16S rRNA genes, and identify the pathogen via a built-in array or sequencer [100]. Such devices, essentially “molecular labs-on-chip”, have been applied to detect pathogens like Mycobacterium tuberculosis, E. coli, and others in low-resource settings. During the review period, there were also demonstrations of CRISPR-based microfluidic diagnostics for infectious diseases: e.g., a chip that combines isothermal amplification with a CRISPR-Cas12 detection step for specific bacterial genes, producing a fluorescent signal that a smartphone camera reads [101]. One recent example is the CHAMP platform by Jing et al. (2024) [102], a centrifugal microfluidic system that integrates CRISPR-Cas12b with real-time LAMP in a single one-pot reaction. This user-friendly “CRISPR on chip” approach (CHAMP) offers high sensitivity and specificity for nucleic acid detection—by adding a fully automated magnetic-bead extraction of nucleic acids into a pre-packaged microfluidic disk, up to 48 samples can be tested simultaneously within 15–60 min [102]. The authors demonstrated the method on 427 clinical nasopharyngeal swab samples for Mycoplasma pneumoniae, with excellent positive/negative predictive values and significant time savings, underscoring how CRISPR-microfluidic integration can accelerate pathogen diagnostics.

Despite these advances, achieving a true “sample-to-answer” microfluidic diagnostic for complex specimens (like whole blood or swabs) remains challenging. Raw clinical samples often contain cells, debris, and inhibitors that require on-chip processing (cell lysis, filtration, plasma separation, etc.) prior to detection. Integrating these steps into a miniaturized device without user intervention is non-trivial. In sepsis testing, for example, a microfluidic chip might need to handle milliliters of blood to capture rare bacteria (often <1 CFU/mL), which necessitates mechanisms to remove or lyse blood cells and concentrate microbes–tasks that can lead to clogs or loss of analyte. Researchers are addressing this by incorporating features like microfluidic filters/membranes, on-chip bead-based DNA extraction, and centrifugo-pneumatic flow control. One approach uses a centrifugal lab-on-disc to perform cell separation and nucleic acid purification from blood, followed by PCR, all in a sealed cartridge. Another strategy employs capillary-driven flow and vacuum-dried reagents in a cartridge to allow “raw sample in, answer out” detection of pathogens from nasal swabs. For COVID-19, devices were developed that accept an unprocessed swab and carry out on-chip viral lysis, RT-LAMP amplification, and CRISPR-based fluorescence readout, with the result read by smartphone—fully integrating sample prep and detection [102]. While such systems show it is possible to minimize manual steps, careful design is needed to prevent cross-contamination and ensure each step (e.g., cell lysis or washing) is as efficient as in a lab. Solutions include novel microvalve networks, pre-loaded dried reagents that dissolve on contact with sample, and sequential flow driven by timers or spinning. As these engineering innovations mature, microfluidic diagnostics are steadily approaching true sample-to-answer capability even for complex samples like whole blood, which would be a major milestone for point-of-care testing in sepsis and beyond.

#### 3.2.3. POC Infectious Disease Panels

Outside of acute care, microfluidic platforms have been applied to routine infectious disease testing in clinics and homes. Examples include HIV viral load testing chips for resource-limited regions (performing on-chip nucleic acid amplification from a fingerstick of blood) and microfluidic immunoassays for diseases like dengue, Zika, or malaria [102,103,104,105,106]. These systems often integrate with portable readers (or smartphones) to provide diagnostic results in settings without full laboratories, supporting outbreak control and surveillance [18]. One noteworthy device is a palm-sized microfluidic cartridge that can simultaneously test for multiple respiratory viruses from a throat swab; it automatically splits the sample into microchambers for individual RT-PCR reactions, and an attached reader reports which viruses are present, all within one hour.

Overall, microfluidic technology has significantly improved infectious disease diagnostics by reducing assay times from days to minutes/hours, enabling on-site testing, and preserving sensitivity and accuracy. The COVID-19 experience demonstrated that microfluidic tests can be mass-produced and deployed globally—millions of cartridge-based tests (some using microfluidic principles) were used in the pandemic [107]. Looking forward, these advances are being generalized to create broad POCT panels for syndromic testing (e.g., one chip for fever illnesses that checks for dozens of pathogens). The marriage of microfluidics with isothermal amplification, CRISPR diagnostics, and smartphone-based readers (often enhanced by AI image analysis, as discussed later) will continue to expand the reach of rapid infectious disease diagnosis.

### 3.3. POCT and Global Health

Microfluidic lab-on-a-chip technology is driving significant advancements in POCT, enabling widespread clinical applications globally. Compared to traditional centralized laboratory methods, microfluidic chips integrate complex analytical procedures into portable devices, offering significant advantages such as portability, rapid testing, and high functional integration [108]. These cost-effective and efficient diagnostic technologies are particularly beneficial in resource-limited settings, where rapid disease identification is critical for reducing disease burden and improving patient quality of life. Recent studies have demonstrated that microfluidic-based POCT devices are deployable in various settings, including pre-hospital emergency care, community clinics, and home environments, providing rapid diagnostics across multiple scenarios. For example, a novel automated microfluidic device can directly process whole blood samples and complete a digital immunoassay for the heart failure biomarker NT-proBNP within 10 min, requiring only 7 μL of blood and achieving a detection limit of 0.94 pg/mL [85]. In a clinical validation study involving 15 serum samples, results obtained by this system exhibited a strong correlation (r = 0.998) with those from the Roche laboratory electrochemiluminescence assay, demonstrating its reliability and clinical feasibility for out-of-hospital heart failure monitoring.

Typical clinical applications of POCT include detecting biomarkers for cardiovascular diseases, metabolic disorders, endocrine conditions, and infections. Significant breakthroughs have been made in the POCT of cardiac biomarkers. Researchers developed a fully integrated microfluidic immunoassay chip that miniaturizes a traditional 96-well ELISA kit into a portable device, enabling ultra-sensitive detection of cardiac troponin I (cTnI) [86]. Equipped with programmable micropumps and valves, this chip performs each step of the ELISA within 15 min using only 30 µL of blood and achieves a sensitivity of 4.88 pg/mL, significantly surpassing standard 96-well methods. Additionally, multiplex microfluidic chips capable of simultaneous measurement of multiple cardiac markers have been developed. One study introduced a spiral microfluidic chip that concurrently detects cTnI, CK-MB, and myoglobin, completing the analysis within minutes and demonstrating rapid screening capability for acute myocardial infarction [87]. In the field of thrombosis and hemostasis, microfluidic POCT uniquely simulates blood flow conditions with minimal whole-blood volumes, enabling rapid assessment of platelet function, thrombin generation, and fibrin formation [109]. Disposable micro-molded chips, paired with compact microscopy and micropump systems, facilitate quick assessment of antiplatelet or anticoagulant medications in emergency conditions such as stroke and intraoperative bleeding.

For metabolic monitoring, portable biochemical POCT devices are now available for glucose, glycated hemoglobin (HbA1c), and kidney and liver function tests. A nano-integrated enzymatic microfluidic electrochemical biosensor enables a one-step serum creatinine assay, automatically eliminating interference and significantly improving performance by integrating gold nanostructures and carbon nanotube electrodes within microfluidic channels [88]. This biosensor shows promise for bedside kidney function assessment, achieving laboratory-level accuracy. Similarly, widely adopted POCT devices for glucose and HbA1c monitoring have achieved clinical laboratory standards in terms of accuracy and stability, significantly facilitating chronic disease management. In reproductive and endocrine health, while traditional urine-based pregnancy tests represent early successes in POCT, newer advanced immunosensors and smartphone-based detection platforms are emerging, capable of quantitatively measuring hormones like progesterone, thus enabling home-based fertility assessments and endocrine monitoring [89].

In the detection of infectious pathogens, multiplex testing is a critical POCT application in global health. The COVID-19 pandemic accelerated rapid deployment of POCT technologies for large-scale screening and surveillance. A notable example is the CRISPR-assisted nucleic acid detection platform mCARMEN, a microfluidic system integrating isothermal amplification and CRISPR-Cas reactions, capable of simultaneously screening for 21 respiratory viruses, including SARS-CoV-2 and influenza variants [82]. Validation studies with over 2000 patient samples demonstrated mCARMEN’s high sensitivity and specificity, aligning closely with genomic sequencing results. Another notable advancement includes a “one-step” portable nucleic acid analyzer, where swabs inserted into the device trigger automatic cell lysis, magnetic bead-based nucleic acid extraction, and multiplex RT-LAMP amplification, simultaneously detecting SARS-CoV-2 and bacterial pathogens in approximately 80 min with detection sensitivities as low as 2.5 copies/µL or 2.5 CFU/µL [110]. These innovations highlight the capability of integrated microfluidic POCT devices to perform multi-pathogen detection onsite without requiring large-scale laboratory equipment, significantly enhancing infectious disease control capabilities [111].

Emerging technological trends in POCT include integration with smartphones and artificial intelligence (AI). Smartphones serve as portable analytical platforms, significantly improving ease and accuracy of result interpretation [112]. For instance, integrating deep-learning algorithms into smartphone apps to read COVID-19 antigen lateral flow tests has markedly improved consistency, particularly in interpreting weak-positive results [94]. In clinical studies involving 1500 training datasets and 135 real-world tests, AI-enhanced systems achieved 98% overall accuracy and maintained above 99% accuracy in low viral load scenarios, significantly outperforming manual interpretation [95]. Furthermore, smartphone connectivity facilitates immediate data upload to cloud platforms, supporting remote healthcare consultations and epidemiological surveillance. The pandemic spurred widespread adoption of cloud-enabled POCT devices, streamlining positive case tracking and public health decision-making. Another critical trend involves new material and platform technologies, such as paper-based assays. These tests utilize capillary action, require minimal external equipment, and are increasingly employed due to their low cost and ease of use, particularly in resource-limited settings [113]. Many COVID-19-specific paper-based diagnostic tests have been rapidly developed for onsite antigen and antibody screening, illustrating practicality despite ongoing efforts to enhance sensitivity and quantification [114]. Researchers are exploring the integration of nanomaterials and electrochemical sensing into paper-based microfluidic assays to further improve performance while maintaining affordability.

From a clinical laboratory perspective, the rapid development of POCT has intensified attention on test reliability and clinical agreement. Rigorous validation studies assessing analytical sensitivity, specificity, and comparability with standard laboratory methods confirm that contemporary POCT devices increasingly match central laboratory standards. Automated microfluidic systems demonstrate exceptional reproducibility and simplified operation, minimizing user error. Regulatory bodies and laboratory professionals continue to develop standardized quality metrics to ensure effective clinical integration. Recent research underscores the significant strides made by microfluidic POCT devices in functionality, accuracy, and clinical feasibility. As smart analysis and connectivity further advance, POCT will increasingly play a pivotal role in global health, fulfilling the vision of accessible, precise, and timely medical diagnostics for all populations.

## 4. Personalized Medicine and Therapeutic Monitoring

Microfluidic technologies are playing an increasingly important role in the era of personalized medicine–tailoring medical treatment to individual characteristics. By handling small samples and even single cells, microfluidic platforms enable analyses that are specific to a patient’s unique disease profile, and they can facilitate ex vivo testing of therapies on patient-derived specimens. In tandem, microfluidic therapeutic monitoring devices are emerging to track patient responses (such as drug levels or biomarker changes) in real time, allowing dynamic adjustment of treatments [115].

### 4.1. Patient-Specific Drug Screening (Tumor-on-Chip)

In oncology, beyond diagnostics, there is a need to determine which therapy a given patient will respond to. Microfluidic “tumor-on-a-chip” models have been developed where a patient’s tumor cells (from a biopsy or CTCs) are cultured in a microfluidic chamber—often in 3D as spheroids or organoids—and exposed to various chemotherapy drugs under conditions mimicking the body [116]. For example, a 2024 study introduced a PDMS–agarose microfluidic device to grow patient-derived tumor spheroids and test anticancer drug responses in parallel channels [90]. By perfusing different drugs through each channel and monitoring spheroid viability (via live-dead fluorescent staining), the chip identified the most effective drug for that patient’s cancer within a few days, essentially a personalized chemosensitivity test. Similarly, digital microfluidic platforms have been applied to precision medicine: one study in 2024 described a digital microfluidic system that dispenses nanoliter droplets containing a patient’s cancer cells and drug compounds, enabling high-throughput screening of many drug combinations on very limited cells [91]. These approaches represent a shift from one-size-fits-all therapy to functional precision medicine, where microfluidics helps pick the right drug by actually testing it on the patient’s cells in vitro.

### 4.2. Organs-on-Chips for Personalized Therapy

Beyond cancer, microfluidic organ-on-a-chip models of tissues (lung, liver, heart, etc.) are being personalized with patient-derived cells (for instance, induced pluripotent stem cell-derived organoids) to study disease and treatment [117]. In 2022, chimeric antigen receptor T-cell (CAR-T) immunotherapy researchers used a microfluidic “body-on-chip” with multiple compartments representing tumor tissue and healthy tissue from a patient, to test CAR-T cell activity and toxicity before actual infusion [118]. Another example is a microfluidic blood–brain barrier (BBB) on chip lined with a patient’s brain endothelial cells, used to evaluate which drugs cross the BBB effectively for that individual [119]. Such personalized organ-chips can inform drug selection and dosing (e.g., if a patient’s liver-on-chip metabolizes a drug too quickly, a higher dose might be needed in vivo). While still largely in research phases, these systems foreshadow a future where personalized drug testing on organs-on-chips could precede and optimize actual treatment.

### 4.3. Microfluidic Single-Cell Omics

Personalized medicine often relies on detailed molecular profiling. Microfluidic devices have enabled single-cell analyses—isolating individual cells and analyzing their genome, transcriptome, or proteome—which is critical in heterogeneous diseases like cancer [120]. High-throughput microfluidic droplet systems for single-cell RNA sequencing (Drop-seq and its successors) were revolutionary. Recent developments aim to make these techniques more clinic-friendly and integrative. For instance, microfluidic chips can now capture single immune cells and perform on-chip library prep to see a patient’s immune repertoire, guiding immunotherapy choices [121]. Additionally, microfluidic devices that sort and analyze exosomes or circulating DNA at the single-vesicle or single-molecule level contribute to personalized diagnostics, as they can detect minor subclones of tumor mutations that indicate emerging resistance, allowing therapy adjustments [122].

### 4.4. Therapeutic Drug Monitoring

Another vital aspect of personalized care is monitoring drug levels and physiological responses to therapy. Microfluidic sensors are being developed for real-time therapeutic monitoring, especially for drugs with narrow therapeutic windows (like chemotherapy, immunosuppressants, anticonvulsants) [123]. One compelling example is a six-layer paper-based microfluidic biosensor for the immunosuppressant drug tacrolimus, used in transplant patients [92]. This disposable device uses a competitive immunoassay within a vertical flow paper stack to measure tacrolimus in a small drop of blood, producing a colorimetric result proportional to drug concentration. The entire test is simple (add blood, wait a few minutes for color to appear) and low-cost, making tacrolimus monitoring feasible outside the lab—important because maintaining the right tacrolimus level is critical to prevent organ rejection without toxicity. Similarly, microfluidic devices for antibiotics (like vancomycin), anti-epileptics, or chemotherapeutics are in development, often employing either immunoassay-on-chip or electrochemical detection to sense drug levels quickly [93].

### 4.5. Wearable and Continuous Monitoring

A frontier in therapeutic monitoring is continuous biosensing of patient parameters. Here, microfluidic technology overlaps with wearable devices. Researchers have created wearable microfluidic patches that sample sweat and measure analytes such as glucose, lactate, or drug metabolites, providing ongoing feedback during exercise or treatment [124]. While many wearables use sensor strips, microfluidics improves sweat collection and analysis. For example, an advanced wearable incorporated a microfluidic network on the skin to collect sweat and an AI-powered sensor to detect stress biomarkers, sending alerts for early dehydration or fatigue [125]. In a therapeutic context, one can imagine a chemotherapy patient wearing a patch that continuously checks an inflammatory marker or white blood cell count via interstitial fluid, warning of neutropenia onset in real time. Some proof-of-concept devices already measure inflammatory cytokines using microneedle-integrated microfluidics on the skin [126].

In essence, microfluidics contributes to personalized medicine by enabling high-resolution analysis of patient samples and on-demand monitoring, which in turn allows treatments to be tailored and adjusted with unprecedented precision. As Huang et al. (2025) note, the combination of microfluidic automation and AI analysis holds great promise to “revolutionize personalized medicine” by optimizing medication dosages and improving disease management for individuals [4]. The continued integration of microfluidic diagnostic data with patient records and AI-driven decision algorithms (e.g., dosage recommendation systems) will further enhance this synergy. However, achieving this vision broadly will require overcoming certain challenges—as discussed in the final sections—including validating these new approaches in clinical trials and ensuring they meet regulatory standards for safety and efficacy.

## 5. Integration of AI in Microfluidic Diagnostics

The fusion of AI with microfluidic technology is a defining trend of the past five years, amplifying the capabilities of lab-on-chip devices in automation, data analysis, and decision support. AI algorithms—particularly those in machine learning and deep learning—can handle the complex, high-dimensional data that microfluidic systems generate (images, multi-sensor signals, large assay datasets) and can also intelligently control device operations. The result is “smart” microfluidic platforms that not only conduct assays but also interpret results and adapt to conditions in real time. Here we outline several key areas where AI has been integrated into microfluidic platforms.

### 5.1. AI for Automated Control and Error Mitigation

Microfluidic diagnostic devices are moving towards fully automated operation, requiring minimal user input. However, one bottleneck has been handling variability in fluid behavior—for instance, formation of air bubbles or incomplete filling of channels can cause test errors. This is where AI-based control has made a significant impact. Research by Bhuiyan et al. demonstrated an AI-controlled microfluidic immunoassay device that uses computer vision to monitor fluid flow and automatically correct issues like bubbles [17]. Their system, operated via a smartphone, employed a trained algorithm to recognize different microfluidic states from the camera feed (e.g., detecting an air bubble in a channel) and then triggered on-chip actuators to resolve the issue (opening a bubble trap to eliminate the bubble). By incorporating this feedback loop, the device achieved highly reliable automated ELISA processing for cardiac troponin I with no user intervention beyond adding the sample. This study is a prime example of AI enhancing robustness: the microfluidic platform could mitigate common failure modes on its own, which is crucial for real-world, unattended operation.

AI has also been used to control droplet microfluidics and digital microfluidics. For example, reinforcement learning algorithms have been trained to optimize the sequence of electrode actuation in a digital microfluidic chip, reducing the time to route droplets and mix reagents in complex protocols. In flow-based chips, machine learning can adjust pump speeds or valve timings on-the-fly to maintain optimal flow conditions. Such adaptive control is particularly useful in long-running assays (like continuous cell perfusion on organs-on-chips) where conditions might drift—the AI controller can tweak parameters to keep the system in the desired state (e.g., maintaining a stable shear stress on cells).

Moreover, AI helps manage hardware limitations of portable controllers. Bhuiyan’s smartphone device, for instance, had limited computational power, so the team developed specialized region of interest cascading algorithms to efficiently process video frames and detect features with minimal central processing unit load. This illustrates how AI can be engineered even into low-power devices for on-chip automation. As a result of such innovations, we are seeing microfluidic platforms that operate as “black boxes”—the user inserts a sample and the device, guided by AI, carries out all steps (mixing, incubation, washing, detection) and produces a result with laboratory accuracy.

### 5.2. AI in Data Analysis and Pattern Recognition

Microfluidic chips often output complex data types: microscope images of cells, fluorescence time-series, multi-channel electrical signals, etc. Interpreting these data to yield a clinically actionable result can be challenging with hard-coded algorithms, especially when results might be subtle or multi-factorial. Here, AI—particularly deep learning—has shown remarkable prowess.

### 5.3. Image-Based AI Analysis

Many microfluidic tests involve imaging, such as counting cells, detecting morphological changes, or reading out fluorescence signals in numerous micro-wells. Convolutional neural networks (CNNs) and other deep learning models excel at image recognition and have been deployed to interpret microfluidic assay images [127]. A notable example is smartphone-based diagnostic platforms that use the phone’s camera to capture images of microfluidic chips and AI to analyze them. Wang et al. (2023) reviewed how such mobile health platforms with image-based AI are enabling detection of molecules, viruses, cells, and parasites in microfluidic POCT devices [18]. For instance, a smartphone app using a deep learning model can analyze a microfluidic blood smear to detect malarial parasites with accuracy rivaling an expert microscopist, or interpret faint test lines on a microfluidic immunoassay that might be difficult for the naked eye [128]. Researchers documented a smartphone microfluidic platform that photographed a fluorescence-based microfluidic assay and used a cloud-based AI to identify COVID-19 positive samples with higher sensitivity than a manual threshold method. By learning from thousands of assay images (both positive and negative), the AI could recognize subtle positive signals and distinguish them from noise or user errors (like partial sample loading) [129].

### 5.4. Signal Processing and Sensor Data

Microfluidic chips with integrated sensors (electrical, optical) produce time-series data that can be complex (e.g., a sum of multiple frequency components or multiple analyte signals overlapping). Machine learning algorithms, including traditional approaches like principal component analysis and newer ones like autoencoders, have been used to deconvolve and classify sensor outputs [130]. One application is in microfluidic electrocardiogram-on-chip or electrophysiology recordings from cells: AI models can filter and interpret these signals to detect anomalies (arrhythmias or neuron firing patterns) [131]. Another is in multi-analyte detection: microfluidic sensor arrays might detect several biomarkers at once, yielding a pattern of signals; AI classification (like support vector machines or neural nets) can take that pattern and determine the disease state. For example, a microfluidic breathalyzer chip that measures multiple gas biomarkers produced a complex signal pattern—a machine learning classifier was trained to identify if the pattern corresponded to a patient with diabetes or lung infection [132]. The AI achieved far better diagnostic accuracy than any single biomarker threshold by exploiting the combined signature.

### 5.5. Droplet and Single-Cell Data

In high-throughput droplet microfluidics, massive data is generated—imaging thousands of droplets per second or tracking cellular responses in each droplet [133]. AI is indispensable for handling this scale. Deep learning has been used to analyze droplet images to detect which droplets contain a single cell or which show a fluorescent reaction product [134]. One group combined droplet microfluidics with deep learning to perform ultra-fast screening of antibiotic-resistant bacteria: a CNNs analyzed videos of droplets containing bacteria and antibiotics to classify each droplet as growth or no-growth, doing so for millions of droplets to output an AST result in ~30 min [84]. Similarly, Yu et al. (2018) [135] demonstrated a deep learning video microscopy method for phenotypic AST, analyzing time-lapse images of freely moving bacteria to determine antibiotic efficacy. Remarkably, their approach could measure drug inhibition effects and derive an MIC within about 30 min, validating results against gold-standard broth dilution. This early example highlights how AI-driven image analysis can drastically shorten AST turnaround times by directly observing bacterial behavior [135].

In all these cases, AI significantly improves diagnostic accuracy and speed. Pattern recognition by AI can uncover subtle features or correlations that human operators or simpler algorithms might miss, thereby increasing the sensitivity of microfluidic assays (fewer false negatives) and specificity (fewer false positives). Importantly, AI can also output confidence metrics, giving clinicians a sense of certainty for each result.

### 5.6. AI Implementation: Data Requirements and Deployment

Deploying AI methods like CNNs in microfluidic image analysis requires careful consideration of training data, computational load, and hardware. Training a robust deep learning model typically demands a large dataset—often on the order of thousands to tens of thousands of images—to capture the variability in biological samples. For example, a CNN that analyzes droplet images for bacterial growth needs extensive training on droplet images labeled as “growth” or “no-growth” to achieve high accuracy. Such training is computationally intensive, usually performed on GPU-equipped workstations or cloud computing clusters. Once trained, however, the model can be compressed or optimized for faster inference on portable devices. There is a trade-off between performing AI analysis on a smartphone versus on the cloud. On-device (smartphone) analysis offers real-time results and data privacy, but mobile processors may struggle with large CNN models. Researchers have addressed this by developing lightweight CNN architectures and leveraging mobile AI accelerators—for instance, recent work demonstrated a smartphone app that uses a simplified CNN to classify colorimetric test results on-chip [136,137]. The inference was fast enough for point-of-care use, though sometimes at the expense of some accuracy or image resolution. On the other hand, cloud-based analysis can employ powerful servers to run more complex algorithms on the microfluidic data, at the cost of requiring wireless connectivity and introducing a slight delay. Rodrigues Moreira et al. (2023) describe an “AI-as-a-Service” architecture for microfluidics, where the smartphone acts mainly as an interface to capture images and upload data to a cloud server that runs a deep learning model and returns the results [94]. This approach offloads computation and allows use of advanced models that might be too slow on a phone. In practice, many smart microfluidic systems use a hybrid approach—performing critical steps like image capture and pre-processing on the device, then either processing locally if the model is small or sending to the cloud if the analysis is too heavy. In summary, successful integration of AI in microfluidics requires not only algorithm development but also engineering around data (collecting large, high-quality training sets) and deployment (choosing between edge or cloud computing). As mobile hardware and network infrastructure improve, we anticipate even complex deep learning models (e.g., for cell morphometry or pathology detection) will be feasible at the point of care, enabling truly smart, autonomous lab-on-chip diagnostics.

### 5.7. Predictive Analytics and Clinical Decision Support

Beyond analyzing raw data, AI can integrate microfluidic results into higher-level clinical decision-making [138]. Because microfluidic devices often yield quantitative measurements of multiple biomarkers, interpreting these collectively to diagnose a disease or predict an outcome can be complex. AI models (like ensemble learners or Bayesian networks) have been built to take multi-marker outputs and provide a diagnostic suggestion or risk prediction [139]. For example, consider a microfluidic sepsis panel that measures several inflammatory cytokines from a drop of blood. An AI-driven decision support system can combine those cytokine levels with patient vitals and perhaps genomic data to predict sepsis severity or recommend a treatment plan, surpassing what any single test could do. Some research prototypes have combined microfluidic immunoassay results with AI to predict the likelihood of cytokine storm in COVID-19 patients, potentially guiding early aggressive therapy for those flagged at high risk [140].

Another arena is prenatal testing: microfluidic chips can perform multiplex analyses on maternal blood (hormones, fetal DNA, protein markers), and machine learning models have been trained to estimate the risk of conditions like preeclampsia or Down syndrome by learning from datasets of known outcomes [141]. The AI effectively learns the “fingerprint” of marker combinations associated with each condition [142]. AI can also incorporate longitudinal data—if a patient uses a microfluidic home test regularly, the AI can detect trends over time (e.g., a slow increase in a tumor marker) and alert to a clinically relevant change [143]. This temporal pattern recognition is important for chronic disease management and is a strength of techniques like recurrent neural networks (RNNs) or time-series classifiers.

One interesting development is the concept of digital twins in healthcare, where a virtual model of a patient is continuously updated with data (including microfluidic test results). AI uses this to forecast disease progression or response to treatments. Microfluidics provides frequent, detailed inputs (like daily biomarker readings), and AI processes them to simulate scenarios—for instance, predicting how a patient’s cancer might respond to a dose change based on microfluidic chemosensitivity assays and current tumor marker trajectories.

### 5.8. AI-Enhanced Design and Development

It’s also worth noting that AI is aiding the development of microfluidic systems themselves (beyond just usage). Machine learning models are used to optimize chip designs (as mentioned earlier) and even to learn from experimental results to suggest new experiments (an aspect of closed-loop or autonomous research). For instance, an algorithm might analyze which microfluidic channel geometry yielded the best cell capture and then propose new geometries to test, accelerating innovation [144]. Additionally, AI-driven modeling can predict how a particular patient’s sample will behave in a microfluidic device (e.g., blood from a leukemia patient might clog a certain chip design due to high cell counts—an AI might flag that and recommend diluting or an alternative design). This predictive approach can inform device customization for certain patient populations.

## 6. Challenges and Future Directions

Microfluidic chip technologies have made remarkable progress, but significant challenges remain for their widespread adoption in clinical practice and global health. Addressing these challenges will be key for scaling up and ensuring regulatory compliance.

### 6.1. Scaling up Manufacturing and Standardization

While advancements in fabrication have improved microfluidic devices, scaling up production while maintaining quality remains a hurdle. Many academic prototypes rely on labor-intensive processes unsuitable for mass production. Future efforts should focus on industrial-scale manufacturing methods like injection molding and high-throughput 3D printing. Standardization of chip formats, dimensions, and fluidic interconnects is essential for broader adoption. Modular microfluidics and standardized test protocols can help achieve this. International standards for microfluidic devices are being developed to facilitate manufacturing, interoperability, and regulatory approval.

### 6.2. Regulatory Approval and Clinical Validation

For clinical integration, microfluidic devices must meet rigorous regulatory standards, demonstrating safety, effectiveness, and clinical utility. This includes proving their accuracy against gold-standard methods and ensuring AI algorithms are unbiased and reliable. Early engagement with regulatory bodies will be critical for defining acceptable validation frameworks. Ongoing clinical trials, such as those for sepsis or cancer detection, are paving the way for future approvals.

### 6.3. Integration into Clinical Workflow

To be adopted widely, microfluidic platforms must seamlessly integrate into existing healthcare systems. Devices must be user-friendly, minimize manual intervention, and ensure minimal risk of error, particularly for POC and home testing. Wireless capabilities and automated data transfer to electronic health records (EHR) will improve workflow integration. Training programs for clinicians will be essential to facilitate the use of these novel platforms.

### 6.4. Material and Sample Compatibility

The compatibility of microfluidic devices with complex biological samples poses a challenge. Issues like protein adsorption or sample clogging can affect device performance. To address this, ongoing research is focused on developing robust, bio-inert materials and chip designs that can handle a variety of patient samples without clogging. Innovations like active cleaning microfluidics and surface coatings are critical to ensuring reliability in clinical settings.

### 6.5. Interdisciplinary Collaboration and Training

Successful implementation requires collaboration among engineers, clinicians, biologists, and regulatory experts. Interdisciplinary teams will help bridge the gap between prototype development and clinical applications. Specialized educational programs in biomedical microfluidics and AI will be necessary to train professionals who can manage both technical and clinical aspects of these systems.

The future of microfluidic chip technologies holds great promise. With advancements in AI, IoT integration, and global health accessibility, these devices could revolutionize diagnostics, providing faster, more personalized care at the point of need. However, overcoming challenges in scaling, standardization, and integration remains essential for realizing their full potential in the healthcare ecosystem.

## 7. Conclusions

Microfluidic chip technologies have evolved into a transformative force in laboratory medicine, enabling faster, more sensitive, and accessible diagnostics. Recent innovations in chip design, fabrication, and sensor integration have led to lab-on-a-chip systems that handle complex clinical assays, such as cancer liquid biopsies and rapid POC infectious disease tests. These devices address critical healthcare needs, including rapid diagnosis, decentralized testing, and personalized treatment monitoring.

The integration of AI has further enhanced microfluidic platforms, optimizing fluidic designs, ensuring reliable operation, and enabling precise data interpretation. AI-driven analysis, combined with the speed and precision of microfluidics, is paving the way for automated diagnostic tools that support real-time clinical decisions. Microfluidics is expanding into new areas, such as early detection of neurological disorders, microbiome profiling, and environmental monitoring. These applications extend beyond the clinical laboratory, highlighting the broad impact of microfluidics in fields like neuroscience and public health. However, challenges remain, including manufacturing scalability, clinical validation, user-friendly design, and integration into healthcare workflows. Overcoming these barriers requires interdisciplinary collaboration between engineers, clinicians, and regulatory experts. Standardization and regulatory advancements are streamlining the path from research to clinical application.

## Figures and Tables

**Table 1:** Advantages of microfluidic chips over traditional laboratory methods.

Feature	Traditional Laboratory Methods	Microfluidic Chip Technology
Sample Volume	Requires milliliters (mL)	Uses nanoliters to microliters (nL to μL)
Processing Time	Hours to days	Minutes to hours
Reagent Consumption	High reagent costs	Minimal reagent usage
Infrastructure	Requires large equipment and trained personnel	Portable, miniaturized, and user-friendly
Accessibility	Centralized laboratories only	Decentralized, POCT

## Data Availability

No new data were created or analyzed in this study. Data sharing is not applicable to this article.

## Abbreviations

The following abbreviations are used in this manuscript:

AI	Artificial intelligence
AST	Antibiotic susceptibility testing
BBB	Blood–brain barrier
CAR-T	Chimeric antigen receptor T-Cell
CNNs	Convolutional neural networks
COC	Copolymers of cycloolefin
CTCs	Circulating tumor cells
EMA	Electronic messaging association
FDA	Food and drug administration
FSH	Follicle-stimulating hormone
HCG	Human chorionic gonadotropin
HER	Electronic health records
HIPAA/GDPR	Health insurance portability and accountability act/general data protection regulation
IoT	Internet of things
ISO	International organization for standardization
LH	Luteinising hormone;
LIS	Laboratory information systems
MEMS	Micro-electro-mechanical system
MIC	Minimum inhibitory concentration
NIST	National institute of standards and technology
PDMS	Polydimethylsiloxane
PMMA	Polymethyl methacrylate
RNNs	Recurrent neural networks
POC	Point-of-care
POCT	Point-of-care testing
RT-LAMP	Reverse transcription loop-mediated isothermal amplification
